# Supplementary material for: Characterization and prediction of clinical pathways of vulnerability to psychosis through graph signal processing
Source: eLife. 2021 Sep 27;10:e59811. doi: 10.7554/eLife.59811 (PMC8476129; doi:10.7554/eLife.59811)
Supplement: Supplementary file 1. [file elife-59811-supp1.docx]

Supplementary File 1: Comparison of severity of CAARMS items at baseline assessment between subjects excluded from the NEURAPRO cohort due to missing data and the rest of the sample.

| Name of CAARMS  item | Number of available data at baseline assessment in excluded subjects  (N=103). | Mean in excluded subjects (N=201) | STD in exclude subjects | Mean in included subjects | Std in included subjects | P-Value of  Difference after FDR correction for multiple comparisons. |
| --- | --- | --- | --- | --- | --- | --- |
| Unusual_thought_cont ent | 103 | 2.3398 | 2.0222 | 2.4925 | 2.0691 | 0.8920 |
| Non-bizarre_ideas | 103 | 3.0971 | 1.7851 | 3.4577 | 1.7145 | 0.4126 |
| Perceptual_abnormalit ies | 103 | 3.2330 | 1.4566 | 3.3333 | 1.5727 | 0.8920 |
| Disorganized_speech | 103 | 1.2330 | 1.2304 | 1.4328 | 1.3516 | 0.5872 |
| Subjective_cognitive_ change | 94 | 2.0319 | 1.2396 | 2.0448 | 1.2260 | 0.9680 |
| Objective_cognitive_c hange | 95 | 0.8526 | 1.0815 | 0.6816 | 0.9209 | 0.5520 |
| Subjective_emotional_ disturbance | 94 | 1.8298 | 1.4565 | 1.9055 | 1.5317 | 0.8920 |
| Blunted_affect | 95 | 1.0737 | 1.3780 | 1.0746 | 1.4175 | 0.9957 |
| Inadequate_affect | 95 | 0.2421 | 0.7816 | 0.2040 | 0.6732 | 0.8920 |
| Alogia | 94 | 0.9149 | 1.1701 | 0.9652 | 1.1806 | 0.8920 |
| Avolition/apathy | 96 | 3.5312 | 1.3761 | 2.9502 | 1.6845 | 0.0985 |
| Anhedonia | 96 | 3.1979 | 1.6709 | 3.1144 | 1.7949 | 0.8920 |
| Social_isolation | 95 | 2.5684 | 1.8022 | 2.6318 | 1.7070 | 0.8979 |
| Impaired_role_functio ning | 93 | 3.8065 | 1.2092 | 3.3682 | 1.5918 | 0.1775 |
| Disorganized_behavio r | 94 | 0.6383 | 1.2080 | 0.4527 | 1.0436 | 0.5520 |
| Aggressive_behavior' | 103 | 2.5922 | 1.4582 | 2.2239 | 1.5377 | 0.3162 |
| Subjective_motor_fun ctioning | 94 | 0.5745 | 0.9999 | 0.4080 | 0.8560 | 0.5520 |
| Objective_motor_func tioning | 95 | 0.1263 | 0.4668 | 0.1194 | 0.5154 | 0.9680 |
| Subjective_bodily_sen sation | 94 | 0.6702 | 1.2563 | 0.5920 | 1.1326 | 0.8920 |
| Subjective_autonomic _functioning | 94 | 1.9362 | 1.4869 | 1.4577 | 1.5651 | 0.1775 |
| Mania | 95 | 0.4000 | 0.9936 | 0.2935 | 0.8476 | 0.6823 |
| Depression | 96 | 3.6771 | 1.1832 | 3.3781 | 1.5054 | 0.4126 |
| Suicidality/self-harm | 103 | 1.8932 | 1.4945 | 1.8010 | 1.5133 | 0.8920 |
| Affective_instability | 93 | 1.6774 | 1.4458 | 1.5771 | 1.5018 | 0.8920 |
| Anxiety | 93 | 3.2043 | 1.4488 | 2.9900 | 1.6822 | 0.6397 |
| OCD | 94 | 1.0000 | 1.4142 | 0.8209 | 1.3519 | 0.6397 |
| Dissociative_symptom s | 94 | 0.7766 | 1.3769 | 0.9502 | 1.2952 | 0.6397 |
| Impaired_subjective_t  olerance_to_normal_st  ress | 92 | 2.2065 | 1.8725 | 2.2388 | 1.8662 | 0.9680 |
