# Supplementary material for: Characterization and prediction of clinical pathways of vulnerability to psychosis through graph signal processing
Source: eLife. 2021 Sep 27;10:e59811. doi: 10.7554/eLife.59811 (PMC8476129; doi:10.7554/eLife.59811)
Supplement: Supplementary file 2. [file elife-59811-supp2.docx]

Supplementary File 2: Comparison of severity of CAARMS items at longitudinal follow-up between subjects excluded from the NEURAPRO cohort due to missing data and the rest of the sample.

| Name of CAARMS  item | Number of available data at follow-up assessment in excluded subjects  (N=103). | Mean in excluded subjects  (N=201) | STD in exclude subjects | Mean in included subjects | Std in included subjects | P-Value of  Difference after FDR correction for multiple comparisons. |
| --- | --- | --- | --- | --- | --- | --- |
| Unusual_thought_cont ent | 12 | 0.3333 | 0.8876 | 0.6219 | 1.3625 | 0.8644 |
| Non-bizarre_ideas | 12 | 1.5000 | 1.7321 | 1.2637 | 1.6838 | 0.8644 |
| Perceptual_abnormalit ies | 12 | 0.9167 | 1.6765 | 1.1940 | 1.7138 | 0.8644 |
| Disorganized_speech | 12 | 0.7500 | 1.3568 | 0.6119 | 1.0671 | 0.8644 |
| Subjective_cognitive_ change | 12 | 0.8333 | 1.1146 | 1.1194 | 1.2065 | 0.8644 |
| Objective_cognitive_c hange | 12 | 0.6667 | 1.3707 | 0.4301 | 0.8574 | 0.8644 |
| Subjective_emotional_ disturbance | 12 | 0.5833 | 1.0836 | 0.7512 | 1.1991 | 0.8644 |
| Blunted_affect | 8 | 1.0000 | 1.4142 | 0.5224 | 1.0774 | 0.8644 |
| Inadequate_affect | 8 | 0.2500 | 0.7071 | 0.1100 | 0.4560 | 0.8644 |
| Alogia | 12 | 0.1667 | 0.5774 | 0.3918 | 0.8229 | 0.8644 |
| Avolition/apathy | 12 | 1.6667 | 1.4975 | 1.4826 | 1.5463 | 0.8644 |
| Anhedonia | 12 | 1.1667 | 1.6422 | 1.1393 | 1.5461 | 0.9813 |
| Social_isolation | 12 | 1.2500 | 1.7123 | 1.2388 | 1.5946 | 0.9813 |
| Impaired_role_functio ning | 12 | 1.0000 | 1.8586 | 1.6020 | 1.8278 | 0.8644 |
| Disorganized_behavio r | 12 | 0.1667 | 0.5774 | 0.1891 | 0.7170 | 0.9813 |
| Aggressive_behavior' | 12 | 1.5000 | 1.4460 | 1.3134 | 1.4718 | 0.8644 |
| Subjective_motor_fun ctioning | 12 | 0.1667 | 0.5774 | 0.0995 | 0.4473 | 0.8644 |
| Objective_motor_func tioning | 11 | 0 | 0 | 0.0500 | 0.4444 | 0.8644 |
| Subjective_bodily_sen sation | 12 | 0 | 0 | 0.1493 | 0.6462 | 0.8644 |
| Subjective_autonomic _functioning | 12 | 0 | 0 | 0.5622 | 1.0897 | 0.8644 |
| Mania | 12 | 0.2500 | 0.8660 | 0.1542 | 0.6173 | 0.8644 |
| Depression | 12 | 1.8333 | 1.1934 | 1.8010 | 1.5749 | 0.9813 |
| Suicidality/self-harm | 12 | 1.0000 | 1.6514 | 0.7910 | 1.2474 | 0.8644 |
| Affective_instability | 12 | 0.6667 | 0.9847 | 0.9701 | 1.3598 | 0.8644 |
| Anxiety | 12 | 1.7500 | 1.6026 | 1.8607 | 1.7206 | 0.9663 |
| OCD | 12 | 0 | 0 | 0.3433 | 0.9574 | 0.8644 |
| Dissociative_symptom s | 12 | 0.1667 | 0.5774 | 0.3980 | 0.9278 | 0.8644 |
| Impaired_subjective_t  olerance_to_normal_st  ress | 12 | 0.5833 | 1.3790 | 1.0249 | 1.5213 | 0.8644 |
