# Supplementary material for: Characterization and prediction of clinical pathways of vulnerability to psychosis through graph signal processing
Source: eLife. 2021 Sep 27;10:e59811. doi: 10.7554/eLife.59811 (PMC8476129; doi:10.7554/eLife.59811)
Supplement: Transparent reporting form [file elife-59811-transrepform.docx]

***eLife’s* transparent reporting form**

We encourage authors to provide detailed information *within their submission* to facilitate the interpretation and replication of experiments. Authors can upload supporting documentation to indicate the use of appropriate reporting guidelines for health-related research (see [EQUATOR Network](http://www.equator-network.org/%20)), life science research (see the [BioSharing Information Resource](https://biosharing.org/)), or the [ARRIVE guidelines](http://www.plosbiology.org/article/info:doi/10.1371/journal.pbio.1000412) for reporting work involving animal research. Where applicable, authors should refer to any relevant reporting standards documents in this form.

If you have any questions, please consult our Journal Policies and/or contact us: [editorial@elifesciences.org](mailto:editorial@elifesciences.org).

**Sample-size estimation**

- You should state whether an appropriate sample size was computed when the study was being designed
- You should state the statistical method of sample size computation and any required assumptions
- If no explicit power analysis was used, you should describe how you decided what sample (replicate) size (number) to use

Please outline where this information can be found within the submission (e.g., sections or figure legends), or explain why this information doesn’t apply to your submission:

The study was conducted on two independent samples of individuals with either genetic (22q11.2 Deletion Syndrome) or clinical vulnerability for psychosis.

The sample of individuals at clinical high risk of psychosis were recruited in the context of a double bling placebo controlled study designed to test the efficacy of Omega 3 Fatty Acids on rates of conversion to psychosis. In this perspective power analyses were conducted resulting in a sample of 320 individuals, sufficient to detect a clinically significant difference in transition rate of approximately 13%. However, in the present manuscript, we did not test for differences across placebo vs Omega 3 treated individuals, considering them as a homogenous sample. In this perspective we did not refer to power calculations as they would, in our opinion, be misleading as to the objective and scope of the manuscript. However, in the methods section describing samples we extensively refer to previous literature describing details of NEURAPRO sample recruitment.

22q11.2 Deletion Syndrome individuals are collected as part of an ongoing longitudinal observational study. Once again, in the present study individuals with 22q11DS were considered as a single homogenous sample. In this perspective no formal power analysis was considered necessary or performed. We did however consider that a final sample size of 57 subjects, is sufficient to reliably estimate Pearson correlations between clinical variables.

**Replicates**

- You should report how often each experiment was performed
- You should include a definition of biological versus technical replication
- The data obtained should be provided and sufficient information should be provided to indicate the number of independent biological and/or technical replicates
- If you encountered any outliers, you should describe how these were handled
- Criteria for exclusion/inclusion of data should be clearly stated
- High-throughput sequence data should be uploaded before submission, with a private link for reviewers provided (these are available from both GEO and ArrayExpress)

Please outline where this information can be found within the submission (e.g., sections or figure legends), or explain why this information doesn’t apply to your submission:

Given the observational nature of the study, data were derived from two baseline and longitudinal assessments, for each of the included subjects. No outliers were excluded. Details regarding sample selection are detailed in the corresponding methods section of the manuscript.

**Statistical reporting**

- Statistical analysis methods should be described and justified
- Raw data should be presented in figures whenever informative to do so (typically when N per group is less than 10)
- For each experiment, you should identify the statistical tests used, exact values of N, definitions of center, methods of multiple test correction, and dispersion and precision measures (e.g., mean, median, SD, SEM, confidence intervals; and, for the major substantive results, a measure of effect size (e.g., Pearson's r, Cohen's d)
- Report exact p-values wherever possible alongside the summary statistics and 95% confidence intervals. These should be reported for all key questions and not only when the p-value is less than 0.05.

Please outline where this information can be found within the submission (e.g., sections or figure legends), or explain why this information doesn’t apply to your submission:

The statistical analysis pipeline is described in the corresponding methods section of the manuscript. Moreover, a graphical overview of the analysis pipeline is provided in Figure 1 and in the corresponding figure legend. Whenever pertinent individual Pearson R coefficients and corresponding exact P values are reported in the original manuscript. Moreover, as supplementary data we report raw Pearson coefficients for each correlation between clinical variables analyzed in symptom networks. Finally, the strength of each individual significant correlation is displayed as color-coding in figures 2, 5 and 6.

(For large datasets, or papers with a very large number of statistical tests, you may upload a single table file with tests, Ns, etc., with reference to sections in the manuscript.)

**Group allocation**

- Indicate how samples were allocated into experimental groups (in the case of clinical studies, please specify allocation to treatment method); if randomization was used, please also state if restricted randomization was applied
- Indicate if masking was used during group allocation, data collection and/or data analysis

Please outline where this information can be found within the submission (e.g., sections or figure legends), or explain why this information doesn’t apply to your submission:

In both 22q11DS sample and NEURAPRO sample individuals were not allocated to separate experimental groups, but were rather considered as homogenous samples.

**Additional data files (“source data”)**

- We encourage you to upload relevant additional data files, such as numerical data that are represented as a graph in a figure, or as a summary table
- Where provided, these should be in the most useful format, and they can be uploaded as “Source data” files linked to a main figure or table
- Include model definition files including the full list of parameters used
- Include code used for data analysis (e.g., R, MatLab)
- Avoid stating that data files are “available upon request”

Please indicate the figures or tables for which source data files have been provided:

As requested, we have uploaded relevant numerical data files that are represented in Graphs and figures.

Specifically, for both 22q11DS and NEURAPRO samples, we have included data files with:

1) Adjacency matrix with Pearson R coefficients providing a numerical representation of the multi-layer symptom network.
2) Loading of symptoms according to the two main network dimensions derived from network dimensionality reduction, employed for spatial embedding of symptoms.
3) Adjacency matrix reporting number of longitudinal paths traversing each network edge and symptoms, required to identify longitudinal network hubs (Gateway symptoms at baseline and Funnel symptoms at longitudinal follow-up).
